# Evaluation of the CT imaging findings in patients newly diagnosed with chronic thromboembolic pulmonary hypertension

**DOI:** 10.1371/journal.pone.0201468

**Published:** 2018-07-30

**Authors:** Alexandra Grosse, Claudia Grosse, Irene Lang

**Affiliations:** 1 Department of Radiology, Medical University of Vienna, Vienna, Austria; 2 Department of Internal Medicine II, Division of Cardiology, Medical University of Vienna, Vienna, Austria; Vanderbilt University Medical Center, UNITED STATES

## Abstract

**Purpose:**

The aim of this study was to evaluate the vascular and parenchymal CT imaging findings, including vessel and cardiac chamber diameter measurements, in patients newly diagnosed with chronic thromboembolic pulmonary hypertension (CTEPH). The CT imaging findings were correlated with hemodynamic measurements and patient outcome.

**Methods:**

Vascular and parenchymal CT findings were assessed on retrospectively ECG-gated MDCT angiography scans in 76 patients newly diagnosed with CTEPH. The diameters of the right and left ventricle (dRV, dLV), the right and left atrium (dRA, dLA), the ascending aorta (dAA), the right and left pulmonary arteries (drPA, dlPA), and the main pulmonary artery (dPA) were measured on axial CT scans. The CT imaging findings were correlated with demographic and hemodynamic data and adverse patient outcome due to right heart failure (RHF).

**Results:**

The majority of patients showed chronic PE, mosaic perfusion, disparity in segmental vessel size, parenchymal densities, bronchial dilatation, and bronchial collaterals in CT. Mean pulmonary artery pressure (mPAP) was not significantly different in patients with and those without chronic PE, mosaic perfusion, disparity in segmental vessel size, parenchymal densities, bronchial dilatation, and bronchial collaterals. Mean PAP showed significant correlations with the CT metrics of dRV/dLV ratio, dRA, dRV, dPA and dPA/dAA ratio, but no correlation with the central thrombi score. By backward linear regression, the dPA/dAA ratio independently correlated with mPAP. Patients who died of RHF tended to have a higher frequency of exclusively chronic peripheral PE and greater dRV/dLV ratios on presenting CT scans compared with survivors.

**Conclusion:**

The majority of patients newly diagnosed with CTEPH show vascular signs of chronic PE, mosaic perfusion, parenchymal densities, disparity in segmental vessel size, bronchial dilatation, and bronchial collaterals on presenting CT scans. Particularly CTEPH patients with exclusively chronic peripheral PE and increased dRV/dLV ratios seem to be at risk of adverse outcome due to RHF.

## Introduction

Chronic thromboembolic pulmonary hypertension (CTEPH) is considered a rare complication of acute pulmonary embolism (PE). CTEPH results from nonresolving thromboemboli that lead to vascular obstruction, accompanied by vascular remodeling in distal nonoccluded pulmonary arteries. In a recent meta-analysis the incidence of CTEPH after acute PE was 3.2% in survivors and 2.8% in survivors without major comorbidities [1]. Pulmonary hypertension (PH) as a result of chronic thromboembolic disease is potentially curable with pulmonary endarterectomy (PEA) surgery [2]. Although newer surgical techniques already allow for resection of thromboemboli at segmental vessel level [2], the presence of organized thrombi in the central pulmonary arteries is generally considered a criterion for operability of CTEPH [3,4]. CT imaging plays a fundamental role in the diagnostic work-up of patients with CTEPH. CT scanning is frequently used to evaluate the location and extent of surgically accessible chronic thromboembolic changes and identify patients with central thromboemboli who are suitable candidates for PEA [2–4]. In addition, preoperative CT imaging can assist in predicting hemodynamic improvement after PEA [3–6], as, for example, the absence of central thrombi and the presence of dilated bronchial arteries on preoperative CT scans were shown to impact the anticipated hemodynamic benefit after PEA [2–4]. Also, CT scanning helps confirm the diagnosis of CTEPH [4,7–11] and can be used to estimate the severity of PH [12–17]. The CT imaging findings in patients with CTEPH have been described in previous reviews [10,18–20], but only a few original reports have thoroughly investigated the CTEPH-related CT features [21–27], and most of these studies were limited to small patient cohorts.

In this study, we comprehensively analyzed the vascular and parenchymal CT imaging findings, including CT based vessel and cardiac chamber diameter measurements, in patients newly diagnosed with CTEPH. Because of the surgical implications and with the aim to investigate the previously raised physiologic hypothesis that the location of chronic thromboembolic occlusion might influence the development of specific CTEPH-related CT features such as dilated bronchial arteries [3,25], vascular signs of chronic PE were divided into (exclusively) central, (exclusively) peripheral and central and peripheral thromboembolic changes. The CT imaging findings were correlated with demographic and hemodynamic data and adverse outcome due to right heart failure (RHF).

## Methods

### Patients

**Fig 1:**
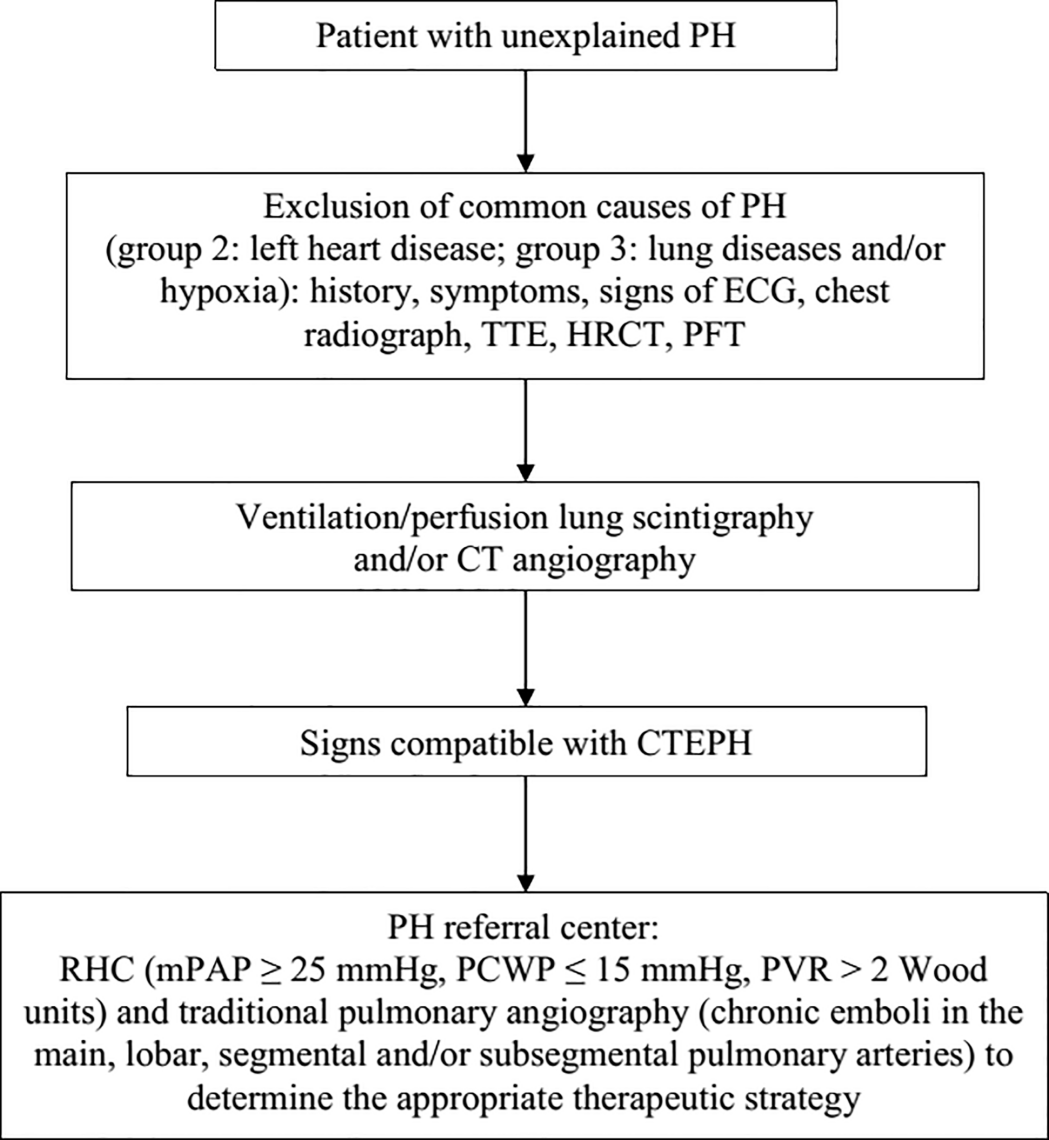
Diagnostic algorithm for chronic thromboembolic pulmonary hypertension according to the European Society of Cardiology (ESC)/European Respiratory Society (ERS) guidelines for the diagnosis and treatment of pulmonary hypertension. CTEPH, chronic thromboembolic pulmonary hypertension; HRCT, high-resolution computed tomography; mPAP, mean pulmonary artery pressure; PCWP, pulmonary capillary wedge pressure; PFT, pulmonary function test; PH, pulmonary hypertension; PVR, pulmonary vascular resistance; RHC, right-sided heart catheterization; TTE, transthoracic echocardiography.

Eighty-one consecutive patients who were referred to our institution for evaluation of newly diagnosed CTEPH between January 2006 and December 2009 were included in this retrospective study. The diagnosis of CTEPH was made based on the results from right-sided heart catheterization, MDCT angiography (MDCTA), pulmonary digital subtraction angiography, and ventilation/perfusion lung scintigraphy in accordance with the 2009 European Society of Cardiology (ESC)/European Respiratory Society (ERS) guidelines for the diagnosis and treatment of PH [28] and its previous version of ESC guidelines [29] (Fig 1). All patients underwent retrospectively ECG-gated MDCTA and pulmonary digital subtraction angiography within 3 months of diagnostic right-sided heart catheterization. Of the initial study population, 5 patients were excluded for the following reasons: time interval of more than 3 months between CT examination and right-sided heart catheterization (*n* = 2), and poor image quality of MDCTA due to insufficient contrast enhancement of the pulmonary arteries or breathing artifacts (*n* = 3). The study was approved by the institutional review board of the University Hospital of Vienna with a waiver of the requirement for informed patient consent.

### CT acquisition

MDCT was performed using a 64-slice CT scanner (Brilliance 64, Philips Healthcare) with 0.5- to 0.625-mm section thickness, 0.5 s/rotation, a tube voltage of 100–120 kV and a tube current of 350–400 mA. The standard CT scan angiographic protocol consisted of administration of 80–85 mL of nonionic contrast agent (iopromide, Ultravist 370, Bayer HealthCare), injected at a flow rate of 4 mL/s and followed by a saline solution flush of 100 mL at the same injection rate. For optimal intraluminal contrast enhancement, automatic bolus tracking was used with the ROI positioned at the level of the ascending aorta and the threshold set at 100 HU for starting data acquisition. CT examinations were performed with the patient in a supine position at suspended deep inspiration. Images were acquired with retrospective ECG-gated scanning and were reconstructed at 75% of the R-R interval with a slice thickness of 1 mm and an increment of 0.8 mm using a standard algorithm. CT images were obtained at mediastinal (window level, 50 HU; window width, 400 HU) and lung (window level, -400 to -700 HU; window width, 1200–1500 HU) window settings.

### Image analysis

**Fig 2:**
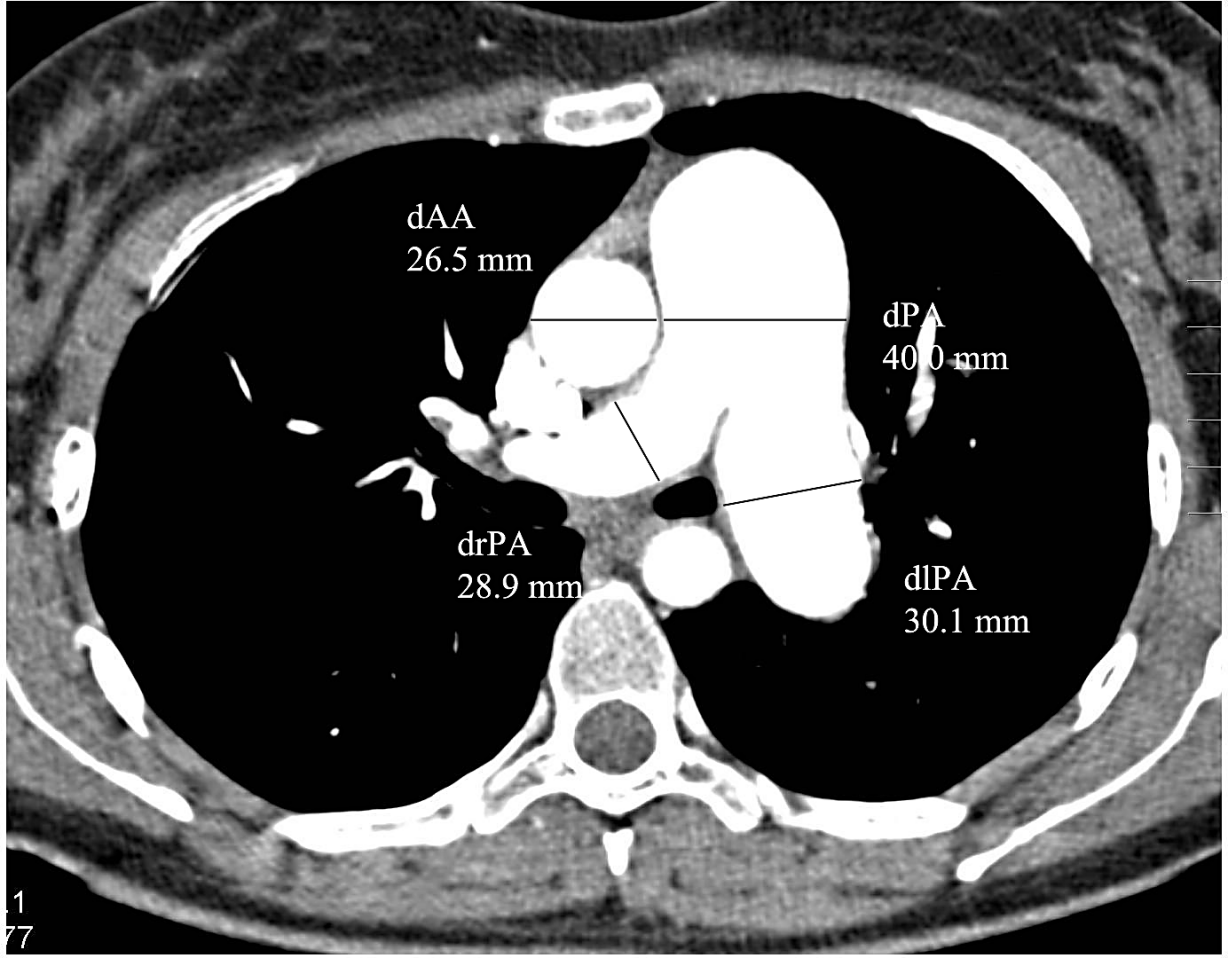
Vascular measurements on MDCTA scans. Main pulmonary artery diameter (dPA), ascending aorta diameter (dAA) and right and left pulmonary artery diameter (drPA, dlPA) were assessed on transverse image.

**Fig 3:**
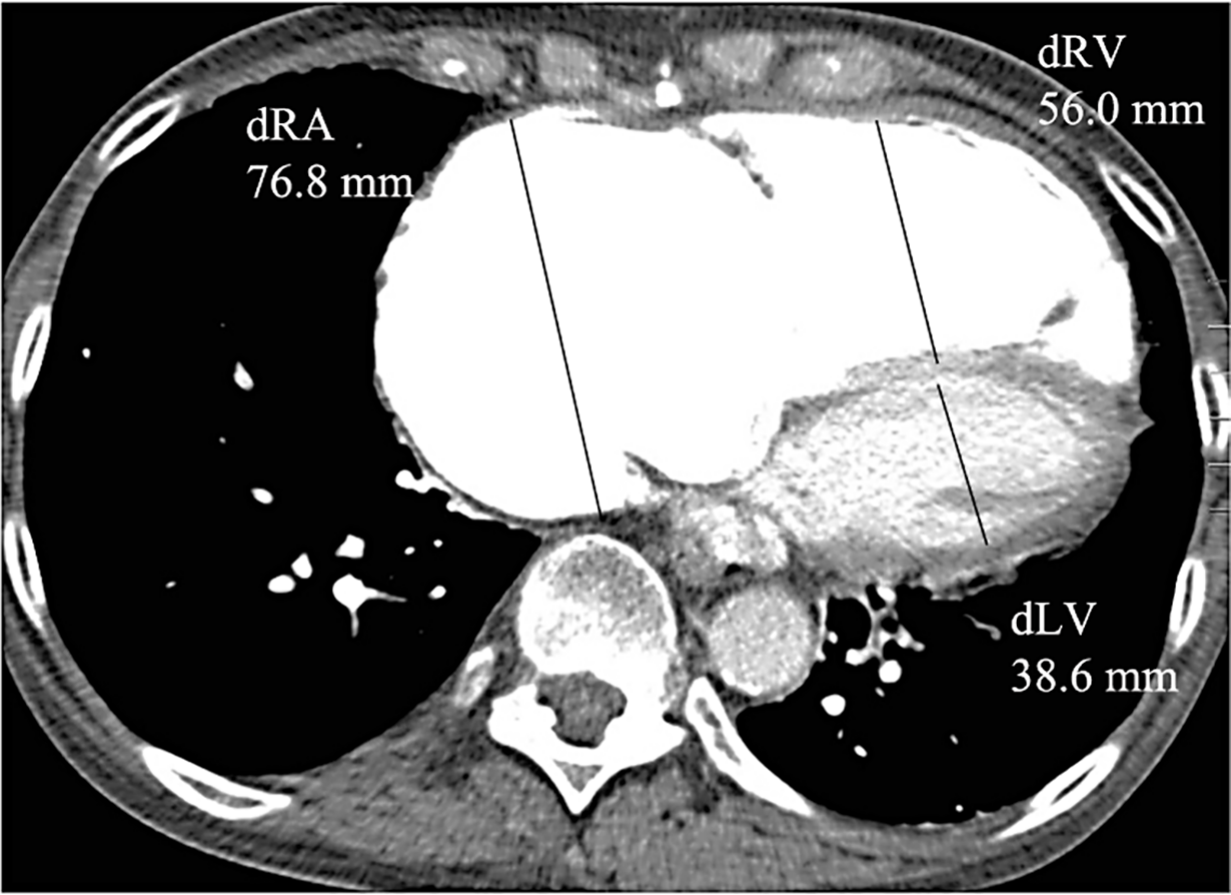
Cardiac chamber size measurements on MDCTA scans. Right and left atrial (dRA, dLA) and ventricular (dRV, dLV) diameters were assessed on transverse image at the end diastole.

The MDCT images were analyzed in random order by two faculty radiologists (with 8 and 13 years of experience, respectively) who were blinded to the patients’ clinical and hemodynamic data, but were aware of the patients’ diagnosis of CTEPH. The widest short-axis diameters of the main pulmonary artery (dPA), the ascending aorta (dAA) and the right (drPA) and left (dlPA) pulmonary arteries were measured on transverse CT sections at the level of the bifurcation of the pulmonary artery trunk (Fig 2), and the dPA/dAA ratio was calculated for each patient. The right and left short-axis atrial (dRA, dLA) and ventricular (dRV, dLV) diameters were measured on axial CT sections at the end diastole (Fig 3), and the dRV/dLV ratio was determined. The MDCTA images at mediastinal window settings were evaluated to assess 1) vascular signs of chronic PE, including wall-adherent thrombi, intraluminal webs or bands, abrupt vessel cutoffs, stenoses, and wall irregularities; 2) dilated bronchial (diameter > 1.5 mm [23]) or non-bronchial systemic (diameter > 4 mm [23]) arteries; and 3) disparity in segmental vessel size. Vascular signs of chronic PE were recorded as central (involving the main and/or lobar pulmonary arteries) or peripheral (involving the segmental and/or subsegmental pulmonary artery branches). For patients in whom segmental vessels were visible at corresponding levels in both the right and the left lungs, the diameters of the segmental pulmonary arteries were measured. Disparity in segmental vessel size was considered to be present when the ratio of the larger vessel to the smaller vessel was greater than 1 [21]. For quantification of the central thromboembolic material we used the Modified Qanadli Index [30], which was shown by Heinrich et al. [3] to be a useful CT score to quantify arterial obstruction in patients with CTEPH. In contrast to the original index, which was introduced to evaluate patients with acute pulmonary emboli [30], we only scored the thromboembolic material in the central pulmonary arteries. The index was defined as patient score divided by the maximal total score and multiplied by 100 (∑[n x d]/40 x 100), where *n* is the number of segmental artery branches distal to the proximal thrombus site and *d* is the severity of obstruction, indicated as 1 for partial obstruction and 2 for complete occlusion [3].

The CT scans at lung window settings were assessed for the presence of mosaic perfusion, parenchymal densities, other parenchymal abnormalities (including emphysema and pulmonary cysts), and bronchial dilatation with or without bronchial wall thickening. The glossary of terms of the Fleischner Society [31] was used for definition and description of the imaging findings. Mosaic perfusion was divided into three patterns: pattern 1, sharply demarcated segmental and/or subsegmental areas of hypo- and hyperattenuation with well-defined borders corresponding to the anatomic unit of the secondary pulmonary lobule; pattern 2, perihilar hyperattenuating areas with peripheral perfusion defects; and pattern 3, diffuse heterogeneity of lung attenuation with patchy low-attenuating areas located more centrally within the secondary lobule and intermixed with areas of normal or increased attenuation.

### Statistical analysis

Descriptive statistics are presented as mean ± standard deviation for continuous variables and as frequency and percentages for categorical variables. Univariate and multivariate analyses were performed to assess the associations of the CT findings with patient data and survival. Univariate analyses were performed by chi-square test or Fisher exact test for categorical variables and by *t* test or nonparametric Mann-Whitney test for continuous variables. Multivariate analysis was performed by multiple linear regression analysis, and a receiver operating characteristic (ROC) curve was drawn to test the ability of dRV/dLV ratio and exclusively chronic peripheral PE to predict RHF-related death in comparison with the prediction according to the full model. *P*-values < 0.05 were considered statistically significant. All statistical analyses were performed with SPSS Statistics software (version 24.0, IBM).

## Results

### Patients

The patients’ demographic and hemodynamic characteristics are reported in Table 1. The study population consisted of 37 men and 39 women (mean age at diagnosis, 56.6 ± 15.5 years; range, 18.1–80.2 years). The average mPAP was 49.9 ± 10.1 mmHg (range, 28 to 73 mmHg). Treatment consisted of specific PH therapy without intervention in 31 (40.8%) patients, PEA in 40 (52.6%) patients and double lung transplantation in 7 (9.2%) patients, including 2 patients who underwent PEA prior to double lung transplantation. Patient follow-up ranged from 6 months to 10.2 years (mean, 4.8 ± 4.0 years; observation cutoff date: May 2017). The majority (75.0%, 57/76) of patients were alive at last follow-up, 15 (19.7%) patients died of RHF within 6 months to 9.3 years (mean, 3.5 ± 4.1 years) of diagnostic CT examination, and 4 (5.3%) patients died of unrelated disease. The mean follow-up for survivors was 5.3 ± 4.0 years (range, 8 months to 10.2 years). The one-year and 5-year survival rates for all patients were 92.1% and 80.3%, respectively.

**Table 1:** Patients’ demographic and hemodynamic characteristics.

	All patients	Survivors	Patients with RHF-related adverse outcome	P-value
Patient Characteristics	(*n* = 76)	(*n* = 57)	(*n* = 15)	
Age (years)	56.6 ± 15.5	57.5 ± 15.9	53.2 ± 15.4	0.347
Sex ratio (M/F)	37/39	28/29	7/8	0.866
mPAP (mmHg)	49.9 ± 10.1	48.5 ± 9.9	54.4 ± 10.8	0.048
Mean follow-up (years)	4.8 ± 4.0	5.3 ± 4.0	3.5 ± 4.1	0.134
Treatment				
Drug therapy	31 (40.8)	24 (42.1)	6 (40.0)	0.883
PEA	40 (52.6)	30 (52.6)	9 (60.0)	0.610
Lung transplantation	7 (9.2)	4 (7.0)	0 (0)	0.291

Data are mean values ± standard deviations for continuous variables and number of patients with percentages in parentheses for categorical variables. mPAP, mean pulmonary artery pressure; PEA, pulmonary endarterectomy; RHF, right heart failure. P-values refer to the comparison between survivors and patients who died of RHF.

### CT imaging findings

**Fig 4:**
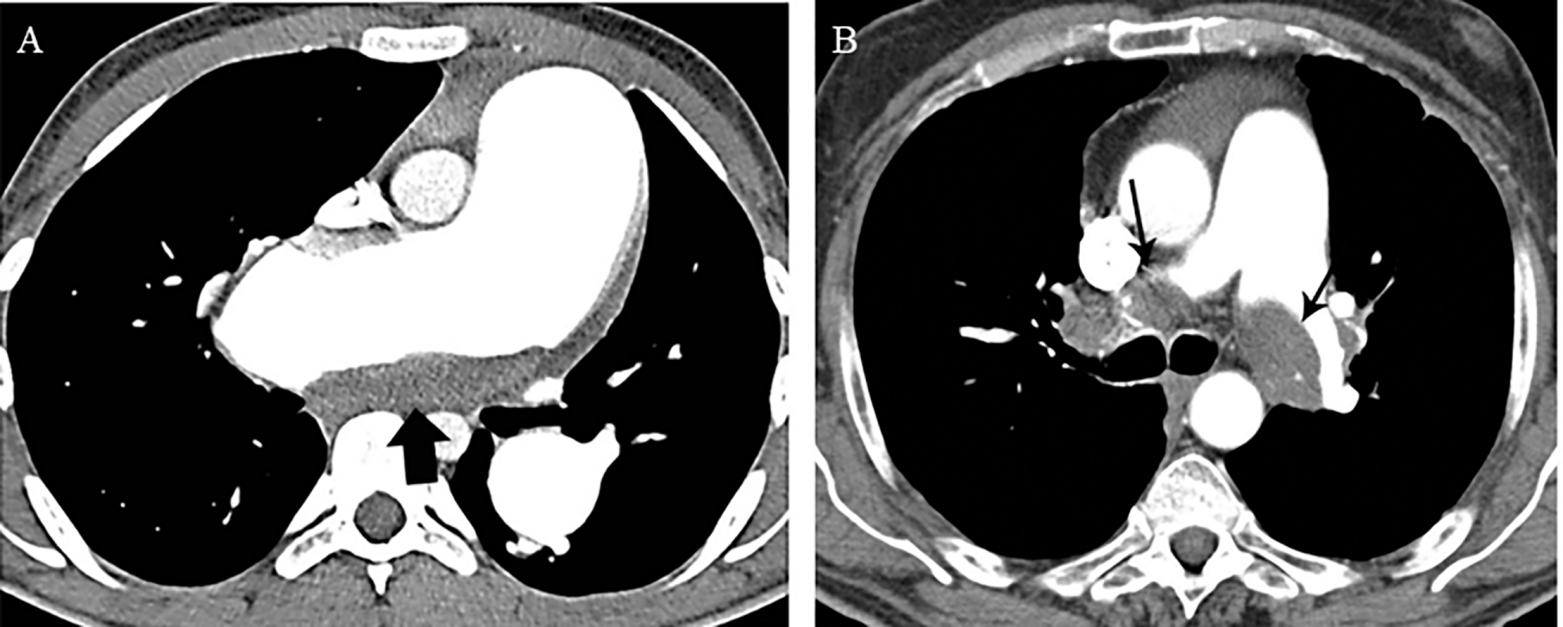
Vascular signs of chronic PE in patients with CTEPH. (A) Axial MDCTA image shows wall-adherent thrombus (arrow) in the pulmonary artery trunk and the right main pulmonary artery. (B) Axial MDCTA image shows wall-adherent thrombus (arrows) in the right and left pulmonary arteries.

**Fig 5:**
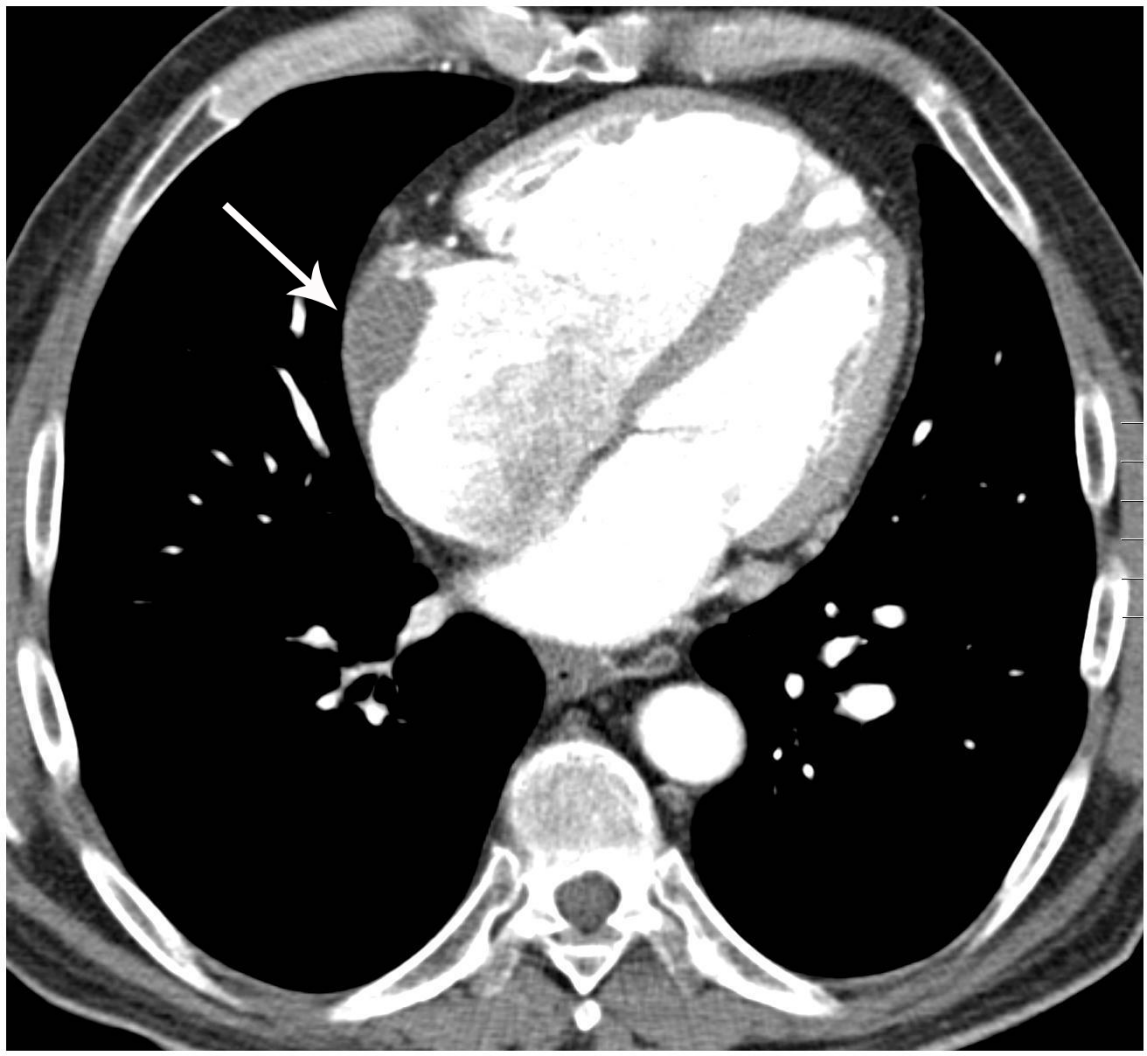
Cardiac thrombi in patients with CTEPH. Axial MDCTA image shows wall-adherent thrombus (arrow) in the right atrium.

**Fig 6:**
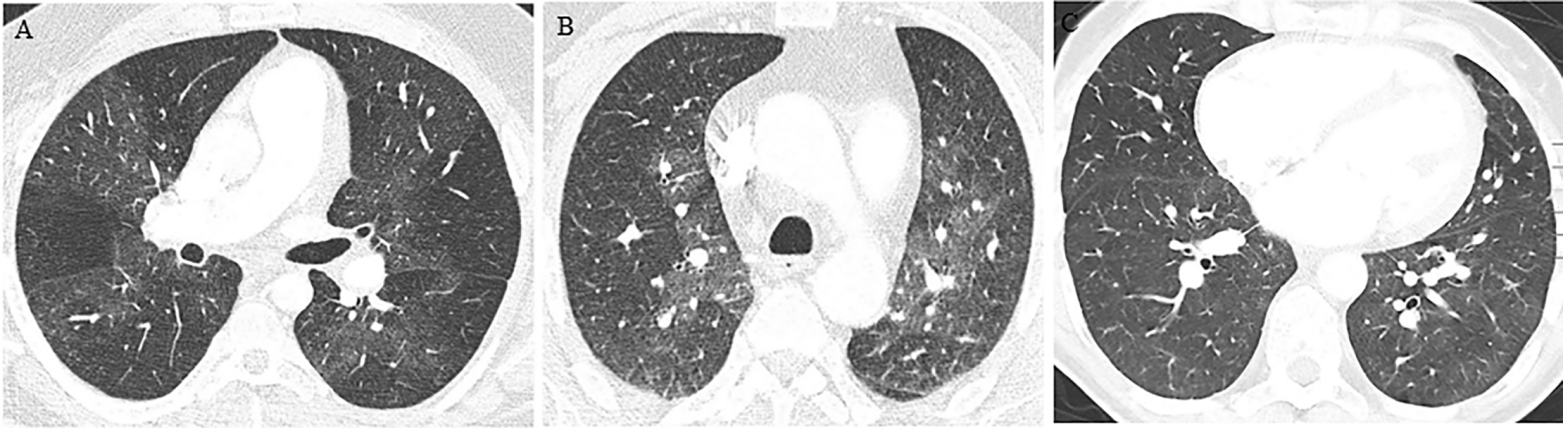
Mosaic perfusion in patients with CTEPH. (A) Axial CT image shows sharply demarcated segmental and subsegmental areas of hypo- and hyperattenuation (pattern 1). (B) Axial CT image shows perihilar areas of increased attenuation and vascularity with peripheral perfusion defects (pattern 2). (C) Axial CT image shows patchy heterogeneous lung attenuation (pattern 3).

**Fig 7:**
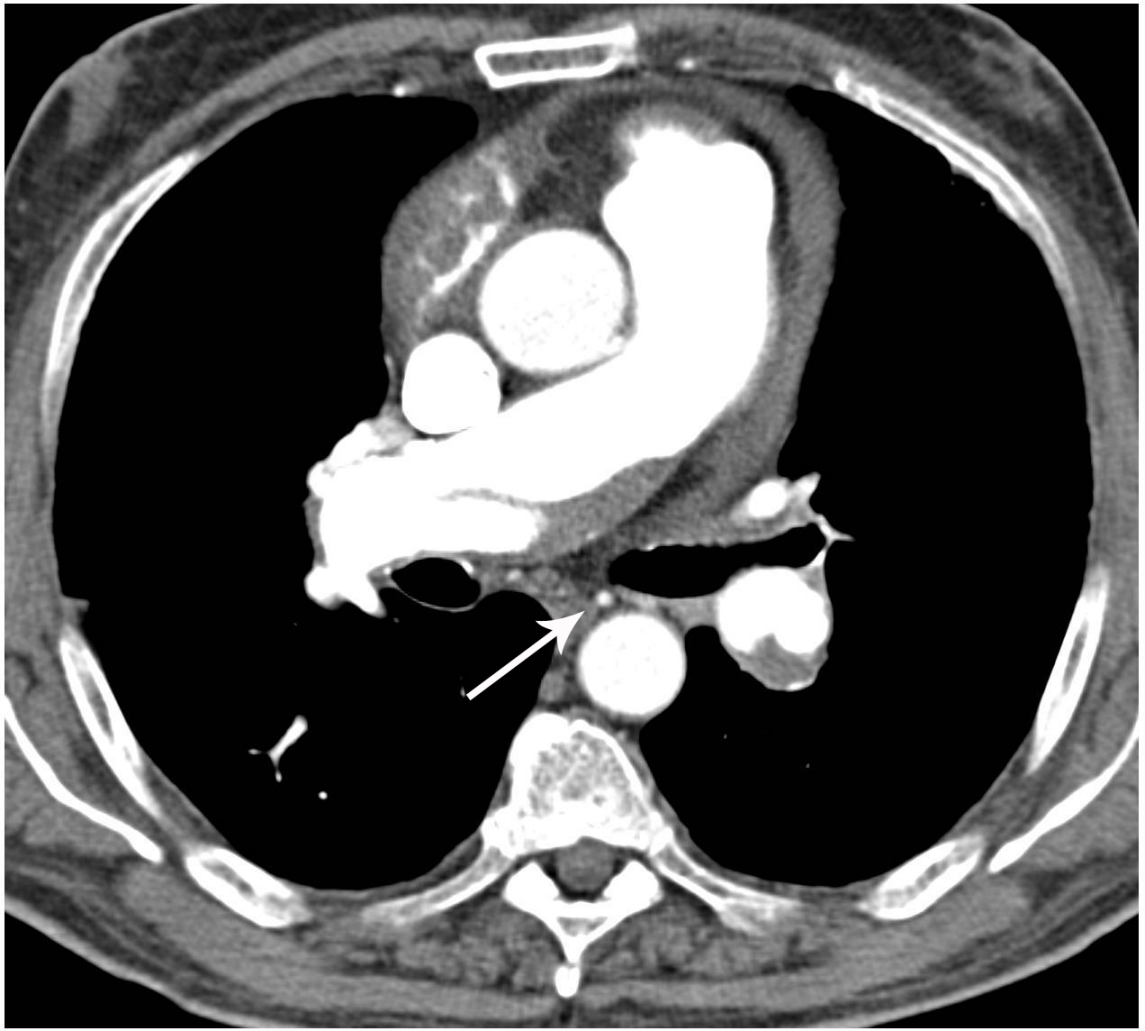
Bronchial collateral arteries in patients with CTEPH. Axial MDCTA image shows bronchial collateral arteries (arrow) in a patient with wall-adherent intraluminal thrombi in the right pulmonary artery and left lower lobe artery.

**Fig 8:**
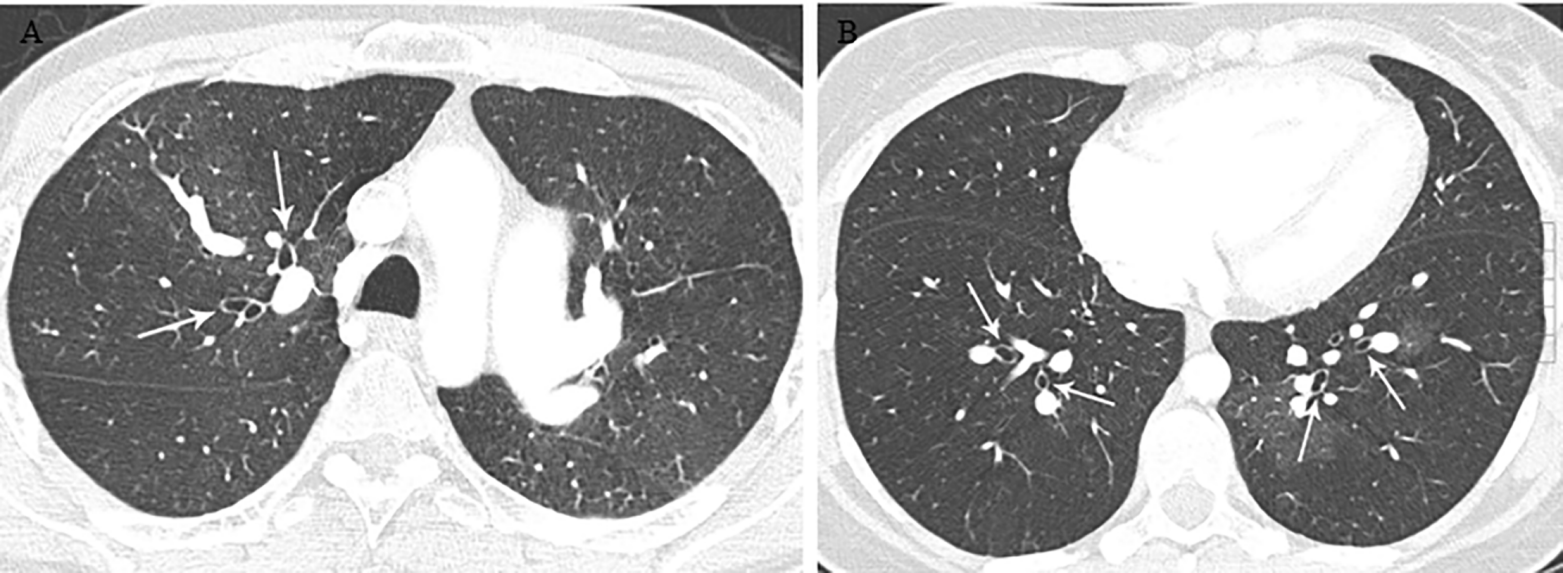
Bronchial dilatation in patients with CTEPH. (A) Axial CT image shows dilated bronchi (arrows) in the right upper lobe. (B) Axial CT image shows dilated bronchi (arrows) in both lower lobes.

**Fig 9:**
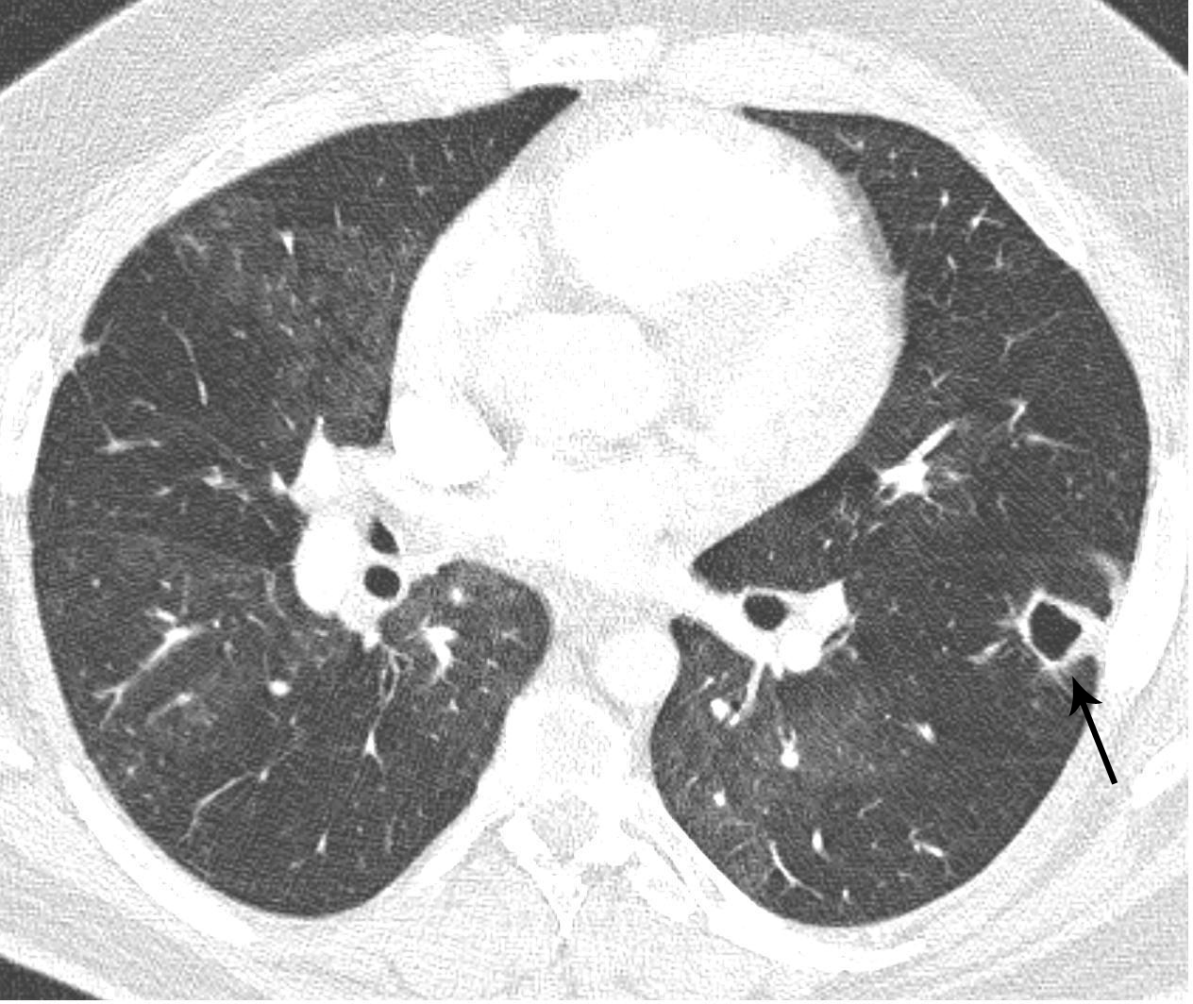
Parenchymal densities in patients with CTEPH. Axial CT image shows peripheral cavitary mass (arrow) combined with mosaic perfusion pattern 1.

The frequencies of the vascular and parenchymal CT imaging findings in the study population are summarized in Table 2. MDCTA scans showed vascular signs of chronic central and/or peripheral PE in 74 (97.4%) of 76 patients (Fig 4). Fifteen (19.7%) patients had exclusively chronic central PE, 23 (30.3%) patients had exclusively chronic peripheral PE, and 36 (47.4%) patients had chronic central and peripheral PE at CT. Acute PE was present at CT in 5 (6.6%) patients in addition to vascular signs of chronic PE, and 2 (2.6%) patients had right atrial thrombi (Fig 5). Disparity in segmental vessel size was observed in 68 (89.5%) patients. The vast majority (70/76, 92.1%) of patients showed mosaic perfusion on CT scans. The predominant pattern, present in 61 (80.3%) patients, was mosaic perfusion pattern 1 followed by pattern 2 in 7 (9.2%) and pattern 3 in 2 (2.6%) patients (Fig 6). CTEPH patients with mosaic perfusion pattern 2 or 3 less frequently displayed disparity in segmental vessel size than patients with mosaic perfusion pattern 1 (4/9 [44.4%] vs 59/61 [96.7%], *p* < 0.001). Bronchial collateral arteries were present in two-thirds (52/76, 68.4%) of patients, and enlarged non-bronchial systemic arteries were identified in almost one-third (24/76, 31.6%) of the study population (Fig 7). Bronchial dilatation and parenchymal densities were found in 61.8% (47/76) and 78.9% (60/76), respectively (Figs 8 and 9).

**Table 2:** CT imaging findings in patients newly diagnosed with CTEPH.

	All patients
Variables	(*n* = 76)
Chronic central and/or peripheral PE	74 (97.4)
Exclusively chronic central PE	15 (19.7)
Exclusively chronic peripheral PE	23 (30.3)
Chronic central and peripheral PE	36 (47.4)
Chronic central PE	51 (67.1)
Wall-adherent emboli	45 (59.2)
Vessel cutoffs	3 (3.9)
Webs, bands, stenoses	9 (11.8)
Chronic peripheral PE	59 (77.6)
Wall-adherent emboli	29 (38.2)
Vessel cutoffs	15 (19.7)
Webs, bands, stenoses	39 (51.3)
Central thrombi score	33.4 ± 14.1
Acute PE	5 (6.6)
Right atrial thrombus	2 (2.6)
Segmental vessel size disparity	68 (89.5)
Mosaic perfusion	70 (92.1)
Pattern 1	61 (80.3)
Pattern 2	7 (9.2)
Pattern 3	2 (2.6)
Parenchymal densities	60 (78.9)
Parenchymal band	50 (65.8)
Wedge-shaped consolidation	8 (10.5)
Round consolidation	8 (10.5)
Cavitary mass	3 (3.9)
Other parenchymal abnormalities	6 (7.9)
Emphysema	5 (6.6)
Pulmonary cysts	1 (1.3)
Bronchial and/or non-bronchial systemic collaterals	55 (72.4)
Enlarged bronchial arteries	52 (68.4)
Enlarged non-bronchial systemic arteries	24 (31.6)
Exclusively enlarged bronchial arteries	31 (40.8)
Exclusively enlarged non-bronchial systemic arteries	3 (3.9)
Bronchial and non-bronchial systemic arteries	21 (27.6)
Bronchial dilatation	47 (61.8)
Bronchial wall thickening	8 (10.5)
With bronchial dilatation	7 (9.2)
Without bronchial dilatation	1 (1.3)
Pleural effusion	9 (11.8)
Pericardial effusion	8 (10.5)

Data are mean values ± standard deviations for continuous variables and number of patients with percentages in parentheses for categorical variables. PE, pulmonary embolism.

The CT-determined measurements of the vessel and cardiac chamber diameters are summarized in Table 3. Seventy (92.1%) of 76 patients had a dPA of ≥ 30 mm, and 31 (40.8%) of 76 patients had a drPA of ≥ 30 mm. The mean dRV was significantly smaller in patients with segmental vessel size disparity than in patients without disparity in segmental vessel size (52.9 ± 10.2 mm vs 61.63 ± 7.0 mm, *p* = 0.014), in the absence of a significant difference in the mPAP between the two groups (50.1 ± 10.4 mmHg vs 48.8 ± 7.7 mmHg, *p* = 0.747). All other CT-determined vessel and cardiac chamber dimensions, including the dRV/dLV ratio, the dPA/dAA ratio and the mean dPA, were not significantly different in patients with and those without chronic (central and/or peripheral) PE, mosaic perfusion, bronchial dilatation, bronchial collaterals, disparity in segmental vessel size, and parenchymal densities (Table 4).

**Table 3:** Vessel and cardiac chamber diameter measurements in patients newly diagnosed with CTEPH.

	All patients
Variables	(*n* = 76)
dRA (mm)	68.2 ± 16.9
dLA (mm)	32.3 ± 6.1
dRV (mm)	53.8 ± 10.2
dLV (mm)	36.5 ± 6.4
dRV/dLV	1.53 ± 0.39
dPA (mm)	37.6 ± 6.1
dAA (mm)	32.6 ± 4.7
dPA/dAA	1.17 ± 0.22
drPA (mm)	28.5 ± 4.2
dlPA (mm)	27.3 ± 3.8

Data are mean values ± standard deviations. dRA, right atrial diameter; dLA, left atrial diameter; dRV, right ventricular diameter; dLV, left ventricular diameter; dPA, pulmonary artery diameter; dAA, ascending aorta diameter; drPA, right pulmonary artery diameter; dlPA, left pulmonary artery diameter.

**Table 4:** Comparison of the CT imaging findings with vessel and cardiac chamber diameter measurements.

CT findings	dRV/dLV ratio	P-value	dPA/dAA ratio	P-value	dPA	P-value
cPE/no cPE	1.53 ± 0.39/1.75 ± 0.43	0.438	1.17 ± 0.23/1.12 ± 0.06	0.753	49.84 ± 10.43/45.50 ± 3.54	0.561
ccPE/no ccPE	1.49 ± 0.36/1.63 ± 0.45	0.158	1.16 ± 0.21/1.20 ± 0.26	0.482	49.98 ± 10.88/49.21 ± 9.27	0.767
cpPE/no cpPE	1.57 ± 0.38/1.42 ± 0.42	0.185	1.19 ± 0.24/1.12 ± 0.14	0.166	50.00 ± 9.90/48.75 ± 11.94	0.672
excl ccPE/no excl ccPE	1.37 ± 0.41/1.58 ± 0.38	0.085	1.12 ± 0.15/1.18 ± 0.24	0.331	49.21 ± 12.72/49.84 ± 9.77	0.839
excl cpPE/no excl cpPE	1.62 ± 0.46/1.50 ± 0.36	0.244	1.21 ± 0.27/1.16 ± 0.20	0.405	49.55 ± 9.60/49.80 ± 10.70	0.924
MP/no MP	1.53 ± 0.39/1.56 ± 0.47	0.884	1.17 ± 0.22/1.21 ± 0.25	0.702	50.21 ± 10.24/43.20 ± 10.06	0.144
BD/no BD	1.47 ± 0.36/1.66 ± 0.42	0.055	1.17 ± 0.21/1.17 ± 0.25	0.964	50.40 ± 11.59/48.59 ± 7.81	0.433
collaterals/no collaterals	1.53 ± 0.38/1.54 ± 0.42	0.951	1.17 ± 0.20/1.18 ± 0.28	0.845	49.74 ± 10.88/49.68 ± 8.82	0.985
brcoll/no brcoll	1.54 ± 0.39/1.53 ± 0.40	0.924	1.16 ± 0.20/1.20 ± 0.28	0.512	49.57 ± 10.99/50.10 ± 8.68	0.845
disparity/no disparity	1.51 ± 0.40/1.74 ± 0.28	0.143	1.16 ± 0.22/1.28 ± 0.27	0.190	49.80 ± 10.56/49.00 ± 8.33	0.847
density/no density	1.51 ± 0.40/1.63 ± 0.37	0.315	1.16 ± 0.21/1.22 ± 0.28	0.505	49.12 ± 10.83/52.21 ± 7.64	0.224

Data are mean values ± standard deviations. dRV, right ventricular diameter; dLV, left ventricular diameter; dPA, pulmonary artery diameter; dAA, ascending aorta diameter; cPE, chronic pulmonary embolism; ccPE, chronic central PE; cpPE, chronic peripheral PE; excl, exclusively; MP, mosaic perfusion; BD, bronchial dilatation; coll, collaterals; brcoll, bronchial collaterals; disparity, disparity in segmental vessel size; density, parenchymal density

### Comparison of the CT imaging findings with demographics, central vs. peripheral location of chronic PE, hemodynamics and RHF

Age and sex ratio were not significantly different in patients with and those without chronic (central and/or peripheral) PE, mosaic perfusion, bronchial dilatation, bronchial collaterals, disparity in segmental vessel size, and parenchymal densities (Table 5). Comparisons of the CTEPH-related CT imaging findings with the location of chronic thromboemboli in the pulmonary arteries showed that patients with chronic central PE were more likely to display disparity in segmental vessel size than patients without chronic central PE (50/51 [98.0%] vs 18/25 [72.0%], *p* = 0.001). Mosaic perfusion occurred with significantly higher frequency in patients with chronic peripheral PE than in those without chronic peripheral PE (57/59 [96.6%] vs 13/17 [76.5%], *p* = 0.007), and patients with mosaic perfusion pattern 2 or 3 less frequently showed chronic central PE at CT than patients with mosaic perfusion pattern 1 (2/9 [22.2%] vs 43/61 [70.5%], *p* = 0.005). By contrast, exclusively chronic peripheral PE was more frequently observed in patients with mosaic perfusion pattern 2 or 3 than in patients with mosaic perfusion pattern 1 (6/9 [66.7%] vs 17/61 [27.9%], *p* = 0.021). Bronchial collateral arteries were significantly more frequent in patients with chronic central PE than in patients without chronic central PE (39/51 [76.5%] vs 13/25 [52.0%], *p* = 0.031), while no significant differences were found in the frequencies of bronchial dilatation and parenchymal densities between patients with and those without chronic (central and/or peripheral) PE.

**Table 5:** Comparison of the CT imaging findings with demographics.

CT findings	Age	P-value	Sex (M/F)	P-value
cPE/no cPE	56.5 ± 15.5/59.3 ± 21.8	0.805	37/37//0/2	0.163
ccPE/no ccPE	57.0 ± 16.0/55.7 ± 14.7	0.749	29/22//8/17	0.042
cpPE/no cpPE	56.4 ± 15.7/57.2 ± 15.3	0.845	31/28//6/11	0.210
excl ccPE/no excl ccPE	56.9 ± 15.2/56.5 ± 15.7	0.916	6/9//31/30	0.453
excl cpPE/no excl cpPE	55.4 ± 14.6/57.0 ± 15.9	0.680	8/15//29/24	0.110
MP/no MP	57.0 ± 15.0/50.9 ± 21.4	0.354	34/36//3/3	0.946
BD/no BD	55.8 ± 14.8/57.9 ± 16.8	0.579	23/25//14/14	0.861
collaterals/no collaterals	55.8 ± 15.0/58.8 ± 16.9	0.458	30/26//7/13	0.154
brcoll/no brcoll	55.8 ± 15.4/58.4 ± 15.8	0.502	28/25//9/14	0.272
disparity/no disparity	57.1 ± 15.7/52.2 ± 13.3	0.401	33/35//4/4	0.937
density/no density	55.9 ± 15.6/58.9 ± 15.3	0.507	29/31//8/8	0.906

Data are mean values ± standard deviations for continuous variables and number of patients for categorical variables. cPE, chronic pulmonary embolism; ccPE, chronic central PE; cpPE, chronic peripheral PE; excl, exclusively; MP, mosaic perfusion; BD, bronchial dilatation; coll, collaterals; brcoll, bronchial collaterals; disparity, disparity in segmental vessel size; density, parenchymal density.

The mPAP was not significantly different in patients with and those without chronic (central and/or peripheral) PE, mosaic perfusion, bronchial dilatation, bronchial collaterals, disparity in segmental vessel size, and parenchymal densities (Table 6). While the central thrombi score did not correlate with mPAP (*p* = 0.877), univariate analyses showed significant correlations of the dRV/dLV ratio (r = 0.32, *p* = 0.006), dRA (r = 0.29, *p* = 0.013), dRV (r = 0.29, *p* = 0.015), dPA (r = 0.39, *p* = 0.028) and dPA/dAA ratio (r = 0.42, *p* = 0.001) with mPAP. Backward linear regression analysis revealed that the dPA/dAA ratio, but not the dRA, dRV, dRV/dLV ratio and dPA, independently correlated with mPAP (model: r^2^ = 0.215, *p* = 0.017; dPA/dAA ratio: beta = 0.274, *p* = 0.025).

**Table 6:** Comparison of the CT imaging findings with mPAP.

CT findings	mPAP	P-value
cPE/no cPE	50.0 ± 10.2/45.5 ± 3.5	0.534
ccPE/no ccPE	50.3 ± 10.6/49.1 ± 9.1	0.632
cpPE/no cpPE	50.1 ± 9.7/49.2 ± 11.7	0.753
excl ccPE/no excl ccPE	49.7 ± 12.4/50.0 ± 9.6	0.937
excl cpPE/no excl cpPE	49.4 ± 9.4/50.1 ± 10.5	0.784
MP/no MP	50.3 ± 10.1/45.5 ± 10.6	0.267
BD/no BD	50.6 ± 11.3/48.8 ± 7.7	0.414
collaterals/no collaterals	49.9 ± 10.7/49.0 ± 8.6	0.991
brcoll/no brcoll	49.6 ± 10.8/50.6 ± 8.4	0.717
disparity/no disparity	50.1 ± 10.4/48.8 ± 7.7	0.732
density/no density	49.2 ± 10.7/52.8 ± 7.3	0.125

Data are mean values ± standard deviations for continuous variables. mPAP, mean pulmonary artery pressure; cPE, chronic pulmonary embolism; ccPE, chronic central PE; cpPE, chronic peripheral PE; excl, exclusively; MP, mosaic perfusion; BD, bronchial dilatation; coll, collaterals; brcoll, bronchial collaterals; disparity, disparity in segmental vessel size; density, parenchymal density.

**Fig 10:**
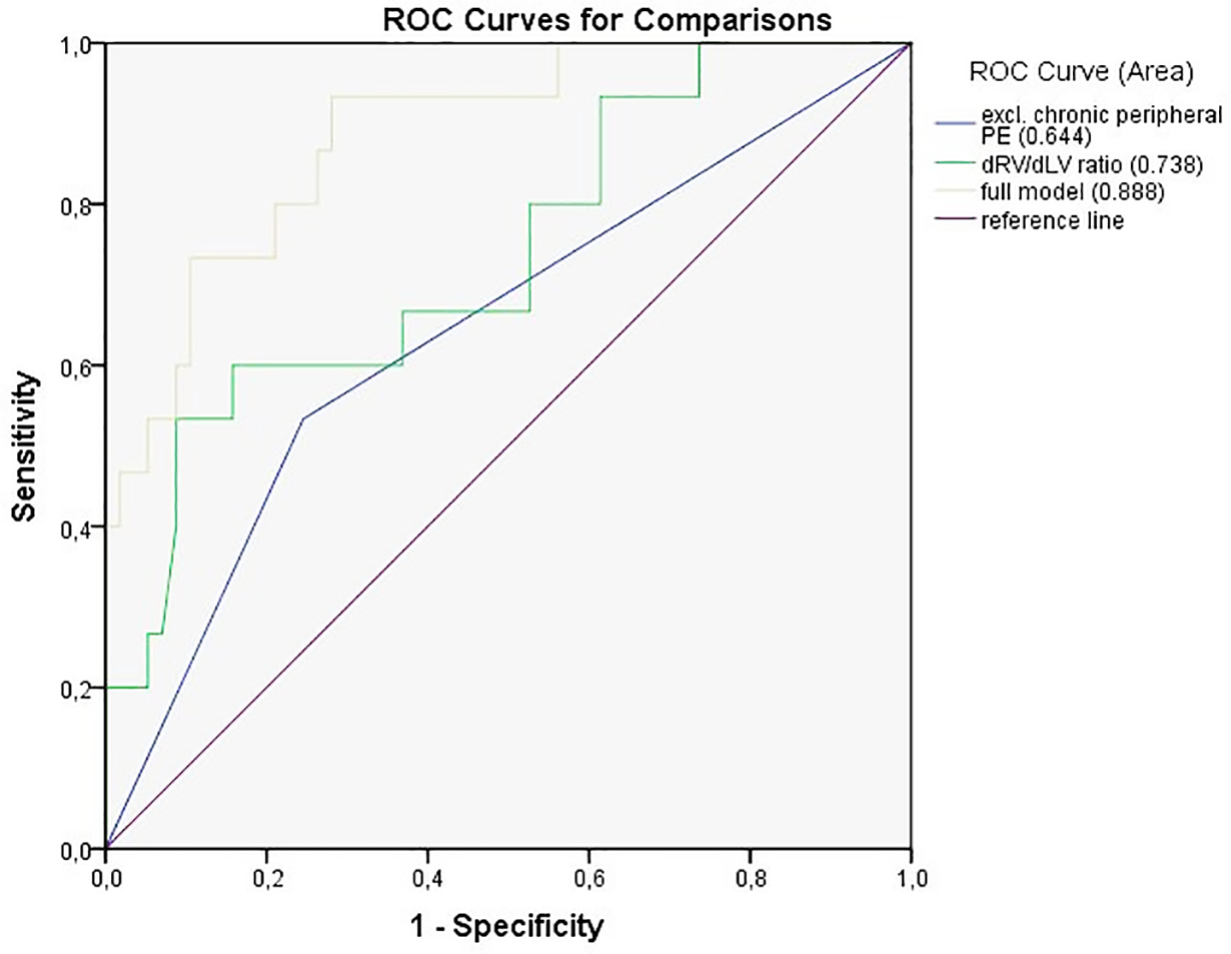
Comparison between ROC curves for RHF-related death prediction according to each significant characteristic with full model.

The frequencies of the vascular and parenchymal CT imaging findings and the vascular and cardiac chamber diameter measurements in survivors and patients who died of RHF are summarized in Tables 7 and 8. Univariate and multivariate analyses showed no significant differences in the mean dPA, the mean dPA/dAA ratio, the central thrombi score and the percentages of chronic (central and/or peripheral) PE, mosaic perfusion, bronchial dilatation, bronchial collaterals, segmental vessel size disparity and parenchymal densities on presenting CT scans between survivors and patients who died of RHF. By contrast, univariate analyses showed that patients with RHF-related adverse outcome had a significantly greater mean dRA (*p* = 0.026), dRV (*p* = 0.044) and dRV/dLV ratio (*p* = 0.002) as well as a significantly higher frequency of exclusively chronic peripheral PE (*p* = 0.031) on presenting CT scans compared with survivors. Multivariate analysis confirmed the significance of these features (dRV/dLV ratio: *p* = 0.021; exclusively chronic peripheral PE: *p* = 0.021), with the exception of dRA and dRV. Fig 10 shows the ROC curves for RHF-related adverse patient outcome (AUC with the full model: 0.888).

**Table 7:** CT imaging findings in survivors and patients who died of RHF.

	Survivors	RHF-related death	P-value
Variables	(*n* = 57)	(*n* = 15)	
Chronic central and/or peripheral PE	55 (96.5)	15 (100)	0.462
Exclusively chronic central PE	11 (19.3)	3 (20.0)	0.951
Exclusively chronic peripheral PE	14 (24.6)	8 (53.3)	**0.031**
Chronic central and peripheral PE	30 (52.6)	4 (26.7)	0.073
Chronic central PE	41 (71.9)	7 (46.7)	0.065
Wall-adherent emboli	37 (64.9)	7 (46.7)	0.197
Vessel cutoffs	3 (5.3)	0 (0)	0.364
Webs, bands, stenoses	7 (12.3)	0 (0)	0.153
Chronic peripheral PE	44 (77.2)	12 (80.0)	0.816
Wall-adherent emboli	23 (40.4)	5 (33.3)	0.620
Vessel cutoffs	11 (19.3)	4 (26.7)	0.532
Webs, bands, stenoses	30 (52.6)	7 (46.7)	0.681
Central thrombi score	32.2 ± 14.2	40.0 ± 14.5	0.187
Acute PE	4 (7.0)	0 (0)	0.291
Right atrial thrombus	2 (3.5)	0 (0)	0.462
Segmental vessel size disparity	52 (91.2)	13 (86.7)	0.596
Mosaic perfusion	53 (93.0)	14 (93.3)	0.962
Pattern 1	47 (82.5)	12 (80.0)	0.826
Pattern 2	4 (7.0)	2 (13.3)	0.758
Pattern 3	2 (3.5)	0 (0)	0.462
Parenchymal densities	48 (84.2)	10 (66.7)	0.127
Parenchymal band	40 (70.2)	9 (60.0)	0.452
Wedge-shaped consolidation	7 (12.3)	0 (0)	0.153
Round consolidation	5 (8.8)	3 (20.0)	0.218
Cavitary mass	3 (5.3)	0 (0)	0.364
Other parenchymal abnormalities	6 (10.5)	0 (0)	0.189
Emphysema	5 (8.8)	0 (0)	0.234
Pulmonary cysts	1 (1.8)	0 (0)	0.605
Bronchial and/or non-bronchial collaterals	42 (73.7)	11 (73.3)	0.978
Enlarged bronchial arteries	40 (70.2)	11 (73.3)	0.811
Enlarged non-bronchial arteries	19 (33.3)	4 (26.7)	0.622
Exclusively enlarged bronchial arteries	23 (40.4)	7 (46.7)	0.659
Exclusively enlarged non-bronchial arteries	2 (3.5)	0 (0)	0.462
Bronchial and non-bronchial arteries	17 (29.8)	4 (26.7)	0.811
Bronchial dilatation	37 (64.9)	8 (53.3)	0.410
Bronchial wall thickening	6 (10.5)	1 (6.7)	0.653
With bronchial dilatation	5 (8.8)	1 (6.7)	0.793
Without bronchial dilatation	1 (1.8)	0 (0)	0.605
Pleural effusion	6 (10.5)	2 (13.3)	0.758
Pericardial effusion	7 (12.3)	1 (6.7)	0.538

Data are mean values ± standard deviations for continuous variables and number of patients with percentages in parentheses for categorical variables. PE, pulmonary embolism; RHF, right heart failure.

**Table 8:** Vessel and cardiac chamber diameter measurements in survivors and patients who died of RHF.

	Survivors	Patients with RHF-	P-value
		related adverse outcome outcome	
Variables	(*n* = 57)	(*n* = 15)	
dRA (mm)	65.7 ± 16.8	76.7 ± 15.8	**0.026**
dLA (mm)	32.1 ± 4.8	32.1 ± 9.5	0.692
dRV (mm)	52.6 ± 10.2	58.4 ± 9.7	**0.044**
dLV (mm)	37.0 ± 5.7	33.3 ± 7.1	0.031
dRV/dLV	1.46 ± 0.35	1.81 ± 0.43	**0.002**
dPA (mm)	37.9 ± 6.3	37.1 ± 6.4	0.665
dAA (mm)	32.9 ± 4.5	31.3 ± 5.6	0.253
dPA/dAA	1.16 ± 0.21	1.21 ± 0.28	0.438
drPA (mm)	28.7 ± 4.6	27.9 ± 3.1	0.641
dlPA (mm)	27.5 ± 4.0	26.8 ± 3.3	0.544

Data are mean values ± standard deviations. dRA, right atrial diameter; dLA, left atrial diameter; dRV, right ventricular diameter; dLV, left ventricular diameter; dPA, pulmonary artery diameter; dAA, ascending aorta diameter; drPA, right pulmonary artery diameter; dlPA, left pulmonary artery diameter; RHF, right heart failure.

## Discussion

In the present study we comprehensively evaluated the vascular and parenchymal CT imaging findings, including CT-determined vessel and cardiac chamber diameter measurements, in patients newly diagnosed with CTEPH. We correlated these findings with baseline mPAP and long-time outcome.

Vascular signs of chronic PE, mosaic perfusion, segmental vessel size disparity, parenchymal densities, bronchial dilatation, and bronchial collateral arteries were found at CT in the majority of patients newly diagnosed with CTEPH. A recent study showed that these CT features occur with significantly higher frequencies in patients with CTEPH than in patients with pulmonary arterial hypertension, PH due to left heart disease, PH due to lung disease or PH due to unknown or multifactorial mechanisms and, therefore, can be used to reliably distinguish CTEPH from other forms of PH [11]. Vascular signs of chronic PE, including wall-adherent intraluminal thrombi, intraluminal webs or bands, abrupt vessel cutoffs, stenoses and wall irregularities, were found in the present study in 97% of patients, and the majority of those had signs of both chronic central and peripheral PE. In a previous high-resolution CT study, Bergin et al. [21] found mosaic lung attenuation combined with segmental vessel size disparity in all 17 patients with CTEPH. However, because the authors did not distinguish between mosaic attenuation due to infiltrative lung or small airways disease and mosaic attenuation due to occlusive vascular disease, the true prevalence of mosaic perfusion in the study population remains unclear. In another investigation, Sherrick et al. [22] described mosaic lung perfusion in 17 (74%) of 23 patients with echocardiographically diagnosed PH due to vascular disease, including 12 (80%) of 15 patients with PH due to PE. However, the authors did not clarify how many patients with CTEPH showed mosaic lung perfusion because the category of PH due to PE included both CTEPH and PH due to acute PE [22]. In the current study, we found mosaic perfusion in 92% of patients newly diagnosed with CTEPH. The most frequent pattern consisted of segmental and subsegmental perfusion defects (pattern 1); a minority of patients showed perihilar hyperattenuation with peripheral perfusion defects (pattern 2) or patchy heterogeneous lung attenuation (pattern 3). Mosaic perfusion pattern 1 was significantly associated with vascular signs of chronic central PE at CT, whereas pattern 2 and pattern 3 were more frequently observed in patients with exclusively chronic peripheral PE. Similarly to the results of a previous study [23], we found dilated bronchial arteries in 68% of patients, and this finding was significantly associated with chronic central PE at CT. Because patients with chronic peripheral or exclusively chronic peripheral PE less frequently demonstrated dilated bronchial arteries, we suggest that the site of thromboembolic vascular occlusion may play a role in the development of pulmonary collateral supply in patients with CTEPH. Parenchymal densities, likely the results of pulmonary infarcts, and bronchial dilatation were found in more than half of the study population, and these findings showed no significant associations with any other CT features evaluated.

In the present study, we found low but statistically significant correlations of the CT metrics of dPA, dPA/dAA ratio, dRV, dRA, and dRV/dLV ratio with mPAP; by backward linear regression, the dPA/dAA ratio was shown to be independently associated with mPAP. Previous studies that investigated the relationship between hemodynamic parameters and vascular CT measurements in patients with PH and controls have demonstrated significant correlations of the CT metrics of dPA and dPA/dAA ratio with mPAP [12–17]. Heinrich et al. [3] analyzed hemodynamic measurements and CT scan findings in 60 patients with CTEPH who underwent PEA. They found significant correlation of dPA with preoperative mPAP (r = 0.42, p < 0.001) but no correlation of dPA with postoperative mPAP. In the same study, the central thrombi score was associated with neither preoperative mPAP nor preoperative pulmonary vascular resistance (PVR) but showed significant correlation with postoperative PVR [3]. Similarly, Liu et al. [32], who retrospectively analyzed hemodynamic and radiologic data of 56 patients with CTEPH, found no significant correlation of CT pulmonary obstruction indexes with mPAP or PVR. However, the authors demonstrated univariate correlations of dRV/dLV ratio, dPA/dAA ratio, dPA, drPA and dlPA with mPAP [32]. In the same study, backward linear regression revealed that the CT metrics of dRV/dLV ratio and dPA were independently associated with mPAP (dRV/dLV, beta: 11.812, p = 0.000; dPA, beta = 0.677, p = 0.003) [32]. In a study including 145 patients with CTEPH, Leone et al. [4] demonstrated significant correlation of mPAP with dPA (p < 0.001) as well as significant associations of mPAP and PVR with severity of mosaic perfusion subclassified into three grades based on a visual scoring system. More recently, Truong et al. [33] reported significant correlation of dPA and dPA/dAA ratio with mPAP and right atrial pressure, using a four-tier severity grading system. Moderate (≥ 31–34 mm) and severe (> 34mm) enlargement of the dPA, and moderate (> 1.0–1.1) and severe (> 1.1) increase of the dPA/dAA ratio were associated with increased mortality as compared to the dPA and dPA/dAA metrics classified as normal [33]. In the current study, patients who died of RHF tended to have a higher frequency of exclusively chronic peripheral PE and a greater mean dRV/dLV ratio on presenting CT scans compared with survivors. As opposed to the results reported by Truong et al. [33], we found no significant associations of baseline dPA and dPA/dAA ratio with mortality. However, when comparing the current study with the study by Truong et al. [33], one must consider the differences in study design and study population between the two investigations: The patient cohort evaluated by Truong et al. [33] was heterogeneous, consisting of 92 non-PH patients and 136 PH patients; during a median follow-up of 6.4 years (5.0–8.2 years) there were 85 deaths, 65 in the PH group and 20 in the non-PH group. By contrast, we exclusively studied patients with CTEPH and correlated CT findings exclusively with death caused by RHF.

Right ventricular function is an important prognostic determinant in predicting survival in patients with CTEPH. Currently, MR imaging is considered the standard of reference in assessing LV and RV function with better reproducibility and accuracy compared with echocardiography [34,35]. In the present study, we used CT imaging to evaluate cardiac chamber dimensions in patients newly diagnosed with CTEPH. CT imaging has some drawbacks in respect of MR imaging such as contrast medium administration and radiation exposure [34]. However, as a result of improved temporal resolution, several studies have recently investigated the role of cardiac CT in providing LV and RV functional parameters, and these studies demonstrated excellent agreement between CT and MR in LV and RV volume assessment [34,36–41]. Thereby, cardiac CT can be regarded as a reliable alternative to echocardiography and MR imaging for patients with poor echocardiographic compliance or with contra-indications to MR [34].

Some limitations of the present study have to be considered. First, this retrospective single-center study comprised a limited number of patients with CTEPH. Prospective multicenter studies are needed with larger patient cohorts to evaluate the potential of CT for identifying high-risk patients with CTEPH and predicting mortality. Second, we compared the imaging findings on presenting CT scans with patient outcome in a heterogeneous patient cohort that received different treatment during follow-up. Hemodynamic changes or alterations of CTEPH-related vascular and parenchymal CT findings as a result of therapy, including modifications of the vessel and cardiac chamber dimensions, were not documented and thus not correlated with RHF and mortality. Future prospective studies are needed to evaluate the relation of CT parameters to hemodynamic outcome, considering patient treatment, and to investigate the role of CT in identifying high-risk patients with CTEPH. Third, although retrospectively ECG-triggered MDCTA more clearly depicts ventricular morphology than helical CT scanning mode, retrospective ECG-gating is currently not recommended as a routine MDCTA protocol because of its higher radiation dose compared with helical scanning mode. Recent studies showed that the use of prospective ECG-gating significantly reduces the radiation exposure to patients undergoing MDCTA and yields diagnostic image quality in the assessment of coronary arteries [42–45], coronary artery bypasses [46], and congenital heart disease [47] with lower radiation dose. Future studies are warranted that evaluate the diagnostic accuracy of low-dose prospectively ECG-triggered MDCTA in patients with CTEPH. Furthermore, because none of our patients underwent conventional angiography of the systemic arteries, the diagnostic accuracy of MDCTA for depicting systemic collateral arteries could not be confirmed with a reference standard. Besides, given the shorter scanning time using modern MDCT technique, it is currently difficult to depict the bronchial circulation, which makes the analysis of the frequency of bronchial collaterals on the CT scans in patients with CTEPH less relevant. Finally, the reviewers were aware of the patients’ diagnosis of CTEPH, which could be a measurement bias, and they used consensus reading for image analysis. Although the consensus reading was based on well-validated criteria for evaluating PH-related CT findings such as dilated bronchial arteries [23,25], bronchial dilatation [24] and mosaic perfusion [22], it is our experience from routine practice that reader variations in the assessment of CTEPH-related CT features may be fairly broad. Thus, the results of our study may not necessarily reflect the observations in routine clinical practice.

## Conclusions

This study showed that vascular signs of chronic PE, mosaic perfusion, parenchymal densities, disparity in segmental vessel size, bronchial dilatation, and bronchial collaterals are frequently observed at CT in patients newly diagnosed with CTEPH. The predominant mosaic perfusion pattern in patients with CTEPH consists of segmental and subsegmental perfusion defects but other perfusion patterns such as perihilar hyperattenuation with peripheral perfusion defects or patchy heterogeneous lung attenuation may occur. In our study, the dPA/dAA ratio correlated with mPAP, while the central thrombi score was not an indicator of severity of PH. The presence of exclusively chronic peripheral PE and an increased dRV/dLV ratio seem to be risk factors of RHF-related adverse patient outcome, but future prospective studies are needed to evaluate the relationship between CT findings and mortality in patients with CTEPH.

## Supporting information

S1 File Patient data. (XLS)

S2 File List of abbreviations. (DOCX)

## Abbreviations

CTEPH: Chronic thromboembolic pulmonary hypertension
ESC: European Society of Cardiology
ERS: European Respiratory Society
MDCT: Multidetector computed tomography
MDCTA: Multidetector computed tomography angiography
mPAP: Mean pulmonary artery pressure
PE: Pulmonary embolism
PEA: Pulmonary endarterectomy
PH: Pulmonary hypertension
PVR: Pulmonary vascular resistance
RHF: Right heart failure
