# Supplementary material for: Evaluation of the CT imaging findings in patients newly diagnosed with chronic thromboembolic pulmonary hypertension
Source: PLoS One. 2018 Jul 30;13(7):e0201468. doi: 10.1371/journal.pone.0201468 (PMC6066236; doi:10.1371/journal.pone.0201468)
Supplement: S2 File — (DOCX) [file pone.0201468.s002.docx]

**Abbreviations**

No – Patient number

RHF – Right heart failure

cPE – Chronic pulmonary embolism

ccPE – Chronic central PE

cpPE – Chronic peripheral PE

excl ccPE – Exclusively chronic central PE

excl cpPE – Exclusively chronic peripheral PE

ccPE+cpPE – Chronic central and chronic peripheral PE

ccPE type 1 – Chronic central PE type 1 (wall-adherent emboli)

ccPE type 2 – Chronic central PE type 2 (vessel cutoffs)

ccPE type 3 – Chronic central PE type 3 (webs, bands, stenoses)

cpPE type 1 – Chronic peripheral PE type 1 (wall-adherent emboli)

cpPE type 2 – Chronic peripheral PE type 2 (vessel cutoffs)

cpPE type 3 – Chronic peripheral PE type 3 (webs, bands, stenoses)

acute PE – Acute pulmonary embolism

MP – Mosaic perfusion

BD – Bronchial dilatation

BWT – Bronchial wall thickening

brcoll – Bronchial collaterals

excl brcoll – Exclusively bronchial collaterals

excl nonbrcoll – Exclusively non-bronchial collaterals

disp – Disparity in segmental vessel size

density – Parenchymal densities

denstype1 – Parenchymal densities type 1 (parenchymal bands)

denstype 2 – Parenchymal densities type 2 (wedge-shaped consolidation)

denstype 3 – Parenchymal densities type 3 (round consolidation)

denstype 4 – Parenchymal densities type 4 (cavitary mass)

dRA – Right atrial diameter

dLA –Left atrial diameter

dRV – Right ventricular diameter

dLV – Left ventricular diameter

dRV/dLV ratio – Right ventricular diameter/left ventricular diameter ratio

dPA – Main pulmonary artery diameter

rPA – Right pulmonary artery diameter

lPA – Left pulmonary artery diameter

dAA – Ascending aorta diameter

dPA/dAA ratio – Main pulmonary artery diameter/ascending aorta diameter ratio

mPAP – Mean pulmonary artery pressure

RA thrombus – Right atrial thrombus

conservative – Conservative treatment

PEA – Pulmonary endarterectomy

LTX – Lung transplantation
